# Multimodal Classification of Parkinson’s Disease in Home Environments with Resiliency to Missing Modalities

**DOI:** 10.3390/s21124133

**Published:** 2021-06-16

**Authors:** Farnoosh Heidarivincheh, Ryan McConville, Catherine Morgan, Roisin McNaney, Alessandro Masullo, Majid Mirmehdi, Alan L. Whone, Ian Craddock

**Affiliations:** 1 School of Computer Science, Electrical and Electronic Engineering, and Engineering Maths, University of Bristol, Bristol BS8 1UB, UK; ryan.mcconville@bristol.ac.uk (R.M.); a.masullo@bristol.ac.uk (A.M.); M.Mirmehdi@bristol.ac.uk (M.M.); ian.craddock@bristol.ac.uk (I.C.); 2 Translational Health Sciences, University of Bristol Medical School, Bristol BS8 1UD, UK; catherine.morgan@bristol.ac.uk (C.M.); alan.whone@bristol.ac.uk (A.L.W.); 3 Movement Disorders Group, Bristol Brain Centre, North Bristol NHS Trust, Bristol BS10 5PN, UK; 4 Department of Human Centred Computing, Monash University, Melbourne, VIC 3000, Australia; roisin.mcnaney@monash.edu

**Keywords:** Parkinson’s disease, deep learning, multimodal data, missing modality, accelerometer, computer vision, variational autoencoder

## Abstract

Parkinson’s disease (PD) is a chronic neurodegenerative condition that affects a patient’s everyday life. Authors have proposed that a machine learning and sensor-based approach that continuously monitors patients in naturalistic settings can provide constant evaluation of PD and objectively analyse its progression. In this paper, we make progress toward such PD evaluation by presenting a multimodal deep learning approach for discriminating between people with PD and without PD. Specifically, our proposed architecture, named MCPD-Net, uses two data modalities, acquired from vision and accelerometer sensors in a home environment to train variational autoencoder (VAE) models. These are modality-specific VAEs that predict effective representations of human movements to be fused and given to a classification module. During our end-to-end training, we minimise the difference between the latent spaces corresponding to the two data modalities. This makes our method capable of dealing with missing modalities during inference. We show that our proposed multimodal method outperforms unimodal and other multimodal approaches by an average increase in $F_{1}$-score of 0.25 and 0.09, respectively, on a data set with real patients. We also show that our method still outperforms other approaches by an average increase in $F_{1}$-score of 0.17 when a modality is missing during inference, demonstrating the benefit of training on multiple modalities.

## 1. Introduction

Parkinson’s disease (PD) is a debilitating neurodegenerative disease with a wide range of motor and nonmotor symptoms, such as slowness of movement, rigidity, tremor, gait dysfunction, posture abnormality, and pain [1]. PD is typically evaluated by specialists in controlled settings, e.g., laboratories or medical centres, where only a snapshot view of the individual’s function can be examined. Parkinson’s symptoms, however, can fluctuate significantly throughout the day, depending on factors such as medication or fatigue levels. Recently, many approaches have been proposed for the automatic assessment of PD [2,3]. Technologies such as the Internet of Things (IoT), which can interconnect multiple sensors in home environments, have extended the potential of these approaches into everyday life [4,5]. Constant collection of sensor data during daily life activities via such technologies could provide an opportunity for continuous monitoring and analysis of PD, and thus provide new insights into the detection and progression of PD. This would not only prevent the evaluation being affected by the fluctuations in the symptoms but also increase its consistency and objectivity by reducing the role of human-based expertise and its inherent subjectivity.

In this paper, we make progress toward automatic “in the wild” PD evaluation in home environments. We utilise an IoT-based platform [6] to collect data from multiple sensors during common activities of daily living. We specifically use camera and wearable inertial measurement unit (IMU) sensors to collect video and acceleration data from PD and healthy control (HC) subjects performing cooking activities in a home environment. Our PD subjects are well-medicated; thus, they show mild symptoms to prevent any inconvenience while cooking. This makes the machine learning task more challenging, but also more realistic. To comply with privacy requirements in home environments [7,8], which are important for real-world use of such a system, we extract per-video-frame silhouettes of the human subjects and then discard all the RGB and depth data. Using these silhouettes, along with the accelerometer data, we propose a multimodal deep learning approach that encodes human movements to discriminate between PD and HC subjects. Note that such distinction between HC and mild well-medicated PD is a difficult task even for clinicians. More specifically, HC subjects can demonstrate impairments consistent with Parkinsonism such as slowness of movement or abnormal posture, which could be due to being elderly [9]. Furthermore, PD is a disease with heterogeneous clinical presentations where one patient may have symptoms that are different from those of the next patient [10]. The challenge of our PD vs. HC classification is magnified by the free-living environment and the cooking task, where the movements and activities are relatively unstructured. In contrast to the detection of specific PD symptoms, however, such general classification of PD vs. HC would consider an impression of the whole body movement in a naturalistic setting, which would be helpful for an automatic diagnosis of PD in its early stages.

Our proposed architecture for multimodal classification of PD (MCPD-Net) is based on the fusion of the two modalities, i.e., silhouette and accelerometer data, via variational autoencoder (VAE) neural networks [11]. Each modality goes through a different VAE network to be reconstructed, while their latent spaces are combined to represent joint features, used for the PD vs. HC classification. Note that such multimodal fusion helps in dealing with the challenges mentioned for the fine-grained distinction between PD and HC in a free-living situation. Compared to a standard unimodal approach, the joint representations learned by MCPD-Net are more robust and effective, as they encode the discriminative information in both modalities and, consequently, reveal different aspects of PD. In particular, the silhouette video data capture the body posture and gait, while the wrist-worn accelerometer records hand movements such as tremors. In our results, we empirically show the effectiveness of these joint representations in recognising PD when compared to single modality features.

MCPD-Net is also capable of handling missing modalities during inference. Note that, in naturalistic settings, modalities may be missing for practical reasons such as the cost of installing vision sensors in every room of the home, technical reasons such as malfunctions, and/or privacy requirements in certain areas of the home. To deal with such missing modalities, we propose to minimise the distance between the latent spaces corresponding to the two modalities. We then use the VAE model of the available modality to generate estimated features for the missing modality. In our results, we show that this approach yields effective representations for the missing modality.

The main contributions of this work are as follows:

- We propose MCPD-Net, a multimodal deep learning model that jointly learns representations from silhouette and accelerometer data.
- We introduce a loss function to allow our model to handle missing modalities.
- We quantitatively and qualitatively demonstrate the effectiveness of our model when dealing with missing modalities, which, for example, due to cost or privacy reasons, is a common occurrence in deployments.
- We evaluate our proposed model on a data set that includes subjects with and without PD, empirically demonstrating its ability to predict if a subject has Parkinson’s Disease based on a common activity of daily living.

The rest of this paper is organised as follows. We first discuss the related works in Section 2. We then explain our proposed method in Section 3. Finally, we present our results and conclusion in Section 4 and Section 5, respectively.

## 2. Related Works

In this section, we first discuss works that evaluate PD using machine learning algorithms. We then discuss multimodal machine learning and approaches that deal with missing modalities.

**Machine Learning for Evaluating PD**—At the heart of research on automatic evaluation of PD, a significant contribution has been made by machine learning algorithms. Many methods have been proposed for diagnostic or progression monitoring purposes, using PD vs. non-PD classification [12,13,14,15,16] or measuring PD symptoms [14,17,18]. In the existing literature, the most commonly used data type is acceleration from smart phones [12,19] or wearable devices [13,15,16,20,21,22]. Some other works also use vision sensors [14,17,18]. Alternatively, some methods for evaluating PD rely on tablets [23,24] or scanner devices [25] for handwriting analysis, or microphones for analysing speech [26,27].

The learning algorithms mainly use raw data or extracted features along with classification methods, such as artificial neural networks (ANN) [12,13,16,20,21,24], random forests (RF) [14,15,19,23,26], support vector machines (SVM) [22,23], and *k*-nearest neighbours (KNN) [26], among others. For example, in [20,21], restricted Boltzmann machines are trained using features extracted from wrist-worn accelerometer data in a home environment to predict PD state. Similarly, [13,16] use convolutional neural networks (CNN) on augmented accelerometer data to classify PD motor state. Li et al. [14] use CNNs on RGB data to first estimate human pose and then extract features from trajectories of joints movements. RF is finally used to classify PD vs. non-PD symptoms and measure their severity. Dadashzadeh et al. [18] also use vision, i.e., RGB and its extracted motion data, to train an end-to-end CNN by which PD symptoms are measured. CNN models are also used on other data types. For example, Taleb et al. [24] use an online handwriting data set to train a deep CNN model for the task of PD classification. Similarly, Gazda et al. [25] train CNN models for detecting PD from offline handwriting.

The works mentioned above report high performance for their learning methods. However, depending on the sensor used, they focus on specific aspects of PD. For example, those using wrist-worn sensors only evaluate PD based on symptoms that are related to hand movements. Likewise, those using vision evaluate PD based on appearance and motion features. In contrast, we propose to use multiple sensors, i.e., cameras and accelerometers, to expand our input domain and capture a wider range of features. In our results, we show that a better performance of PD vs. HC recognition is achieved by fusing the two data modalities, compared to individual ones. Moreover, while vision has proved to be a powerful modality for evaluating PD, privacy issues in home settings has limited research on RGB data. To deal with this, we reduce the means for identification by taking an approach similar to [28,29], in which human silhouettes are extracted and RGB and depth data are discarded.

**Multimodal Machine Learning**—There is a long history of research in this area, exploring different directions [30,31,32]. Representation learning [33,34,35] is one of such directions in which effective and robust joint features are learned, typically from large-scale data sets, to be used in general downstream tasks, such as visual question answering or visual commonsense reasoning. Multimodal fusion [36,37,38] is another major topic in multimodal learning that addresses predefined tasks, such as sentiment analysis, action recognition, image translation, and semantic segmentation, by designing specific architectures for integrating the multiple input modalities.

Despite the variety of architectures, existing multimodal networks are mostly designed for combining vision and language and, less frequently, audio [39,40]. For example, refs. [33,34] use transformer-based models to discover the inherent semantic correlations between vision and language. However, a relatively small part of research in the multimodal learning literature deviates by focusing on other data types such as vision and body-worn IMU data [28,29,41,42], where the modalities are mainly correlated due to the body movements of the subjects. Among these works, [28] proposes a network, called CaloriNet, for fusing accelerometer and silhouette data to estimate the calorie expenditure of the subjects. We find [28] particularly relevant to our work, not only due to their similar input modalities, but also their health-related objectives. As PD affects the activity level of the patients and, consequently, their energy consumption, CaloriNet would be also expected to perform well in discriminating between PD and HC. In our results, we compare the performance of our proposed method with [28] on the task of PD vs. HC recognition.

**Missing Modalities**—Some works in the literature fuse multimodal data, while particularly considering imperfect or missing modalities [43,44,45,46,47,48]. Among them, some use the generative capability of VAE models [44,45,46,48]. For example, Suzuki et al. [44] use a VAE model to present a joint latent distribution of multimodal data. To deal with a missing modality during inference, they also train unimodal VAEs, predictions of which are penalised for their difference with the joint latent distribution. Wu and Goodman [45] also predict a joint latent distribution using a product-of-experts network, which multiplies the unimodal distributions. In addition, to simulate the situation of missing modalities during inference, they take a training regime, in which subsets of modalities are randomly sampled to be used in the VAE optimisation objective. In a similar approach, Shi et al. [48] compute the joint posterior as a mixture-of-experts, i.e., an average over the unimodal latent distributions. The joint model is evaluated using samples from modality-specific latent distributions and, finally, the resulting losses are also averaged.

Similar to these works, we also use VAE models to combine multiple modalities considering missing data. However, our work is different in two ways. Firstly, the VAE models in the mentioned works are trained and assessed for their reconstruction performance. Our goal, in contrast, is classification between PD and HC. Our VAE models mainly aim to predict effective representations for such classification. We thus train the classification and VAE models together end-to-end. Secondly, the mentioned works consider the data and its labels, captions, or attributes as different input modalities. As these basically represent the same entities in different domains, the joint embeddings learn to capture their semantic correlation. In our approach, however, the two modalities inherently represent two different data types, the correlation of which is due to, for example, the patterns in the subjects’ movements. Hence, in our architecture, we predict the two modality embeddings independently to be then fused for classification.

## 3. Materials and Methods

**Figure 1:**
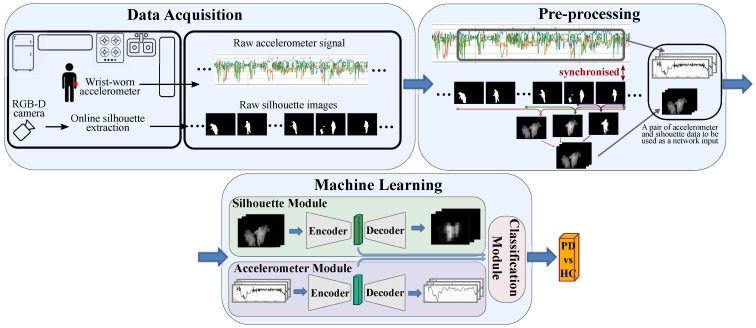
The overall scheme of our proposed approach for classifying PD vs. HC. First, data are recorded in a kitchen while the participant is cooking. They are then preprocessed to be given to the proposed machine learning algorithm for classification.

We now present our proposed approach for recognising PD vs. HC with its overall scheme shown in Figure 1. We define three main phases for our approach. First, we capture data from a camera and an accelerometer device, while the participant performs cooking activities in a kitchen. The RGB-D camera extracts the silhouette data online, which, along the accelerometer signal, go through a preprocessing phase. This is where the input to the last phase, i.e., the machine learning algorithm, is constructed. While the data set specifications (phase 1) are explained in Section 4.1, in this section, we focus on how the network input is formed (phase 2) and the network architecture (phase 3).

**Figure 2:**
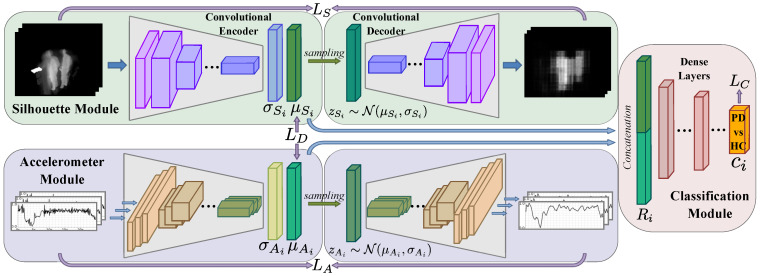
MCPD-Net: our proposed network consists of three modules: silhouette, accelerometer, and classification. Representations learned in the two former modules are fused in the latter module, for PD vs. HC classification.

MCPD-Net, illustrated in Figure 2, consists of three modules, namely silhouette, accelerometer, and classification modules. The silhouette and accelerometer modules are VAE models that learn effective embeddings while reconstructing their two input modalities. These embeddings are then combined using the classification module to predict PD and HC labels. The whole network is trained end-to-end.

**Silhouette Module:** This is a VAE model that reconstructs its input to learn discriminative features from silhouette images, each of which is corresponding to one RGB-D video frame. We generate these silhouettes from RGB-D images, using the method from Hall et al. [49], which applies a combination of background subtraction and the OpenNI library [50].

The input is then temporally encoded by stacking temporally averaged silhouette images using different time scales (as in [28]). More specifically, consider a set of binary silhouette images, $S=\{S_{i}\in {\left\{0,1\right\}}^{H\times W}\;|\;i\in \left\{1,\ldots ,N\right\}\}$, where *N* is the number of the silhouette images in the training set and *H* and *W* represent their height and width, respectively (the same silhouette temporal encoding approach is also applied for the test set). The set of silhouette inputs to the network is then defined as $I_{S}=\left\{I_{S_{i}}\in {\left[0,1\right]}^{H\times W\times D}\;|\;i\in \left\{t_{D},\ldots ,N\right\}\right\}$, where *D* is the depth of each silhouette input $I_{S_{i}}$, and,

(1) $$I_{S_{i},\left(:,:,d\right)}=\frac{1}{t_{d}}\sum_{j=i-t_{d}+1}^{i}S_{j},$$

with $t_{d}\in \left\{t_{1},\ldots ,t_{D}\right\}$ representing the time interval corresponding to the depth channel *d*, where d∈{1,…,D}. Thus, $I_{S_{i}}$ is computed as a 3D tensor made of *D* channels, where its *d*th channel represents the average of $S_{i}$ and its previous $t_{d}$ silhouette images.

**Figure 3:**
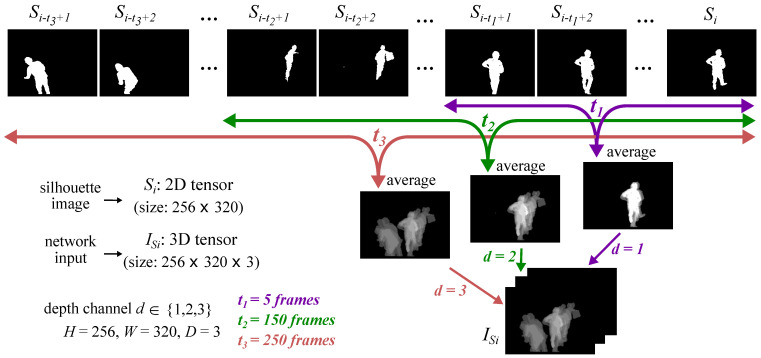
Illustration of the temporal encoding for the silhouette images according to Equation (1). The three depth channels of the network silhouette input $I_{S_{i}}$ consist of averaged images over 5, 150, and 250 frames, respectively.

Figure 3 illustrates this approach for an example with three depth channels, i.e., D=3, and time interval $t_{d}$ equal to $t_{1}=5$, $t_{2}=150$ and $t_{3}=250$ silhouette frames (These numbers match our implementation settings in Section 4.2). Note that the minimum *i* index here equals $t_{D}=250$. This means that the first silhouette input is $I_{S_{250}}$, generated from $S_{250}$ and its 5, 150, and 250 previous silhouette frames, respectively.

This method is capable of encoding the mobility and posture of the subjects over time, and thus encapsulates discriminative features for recognising PD. Moreover, this method has shown effective performance in encoding silhouette images as binary entities [28].

The silhouette input $I_{S_{i}}$ is then given to a convolutional VAE, which outputs $O_{S_{i}}$ as follows:

(2) $$\begin{array}{cc} \mu_{S_{i}},\sigma_{S_{i}}= & \;e_{S}\left(I_{S_{i}};\theta_{e_{S}}\right),\\ z_{S_{i}}∼ & \;N(\mu_{S_{i}},\sigma_{S_{i}}),\\ O_{S_{i}}= & \;d_{S}\left(z_{S_{i}};\theta_{d_{S}}\right), \end{array}$$

where $e_{S}$ and $d_{S}$ represent the encoder and decoder networks of the silhouette VAE, and $\theta_{e_{S}}$ and $\theta_{d_{S}}$ are their parameter sets, respectively. According to Equation (2), the encoder $e_{S}$ outputs the parameters of a normal distribution, i.e., its mean $\mu_{S_{i}}$ and covarince $\sigma_{S_{i}}$, from which a latent representation, i.e., $z_{S_{i}}$, is sampled and given to the decoder $d_{S}$. The silhouette VAE is then optimised by minimising the reconstruction loss and the Kullback–Leibler (KL) divergence between the distribution parametrised by the encoder outputs and a standard normal distribution,

(3) $$L_{S}=\sum_{i}(∥O_{S_{i}}-I_{S_{i}}{∥}^{2}+KL\left(N\left(\mu_{S_{i}},\sigma_{S_{i}}\right),N\left(O,I\right)\right)).$$

**Accelerometer Module:** This is also a VAE model that processes the accelerometer time series data. Similar to [28], the lengths of the accelerometer input sequences are set to the maximum time interval ($t_{D}$) used for our silhouette inputs. More specifically, $I_{A}=\left\{I_{A_{i}}\in R^{t_{D}\times 3}\;|\;i\in \left\{t_{D},\ldots ,N\right\}\right\}$ represents the set of accelerometer sequences, where their first dimension represents time and their second dimension represents the three spatial directions of the acceleration signal (*x*, *y*, *z*). Note that each $I_{A_{i}}$ corresponds to $I_{S_{i}}$ from Equation (1), i.e., they are both given to the network as an input pair.

Similar to the silhouette module, the accelerometer input $I_{A_{i}}$ is given to the VAE model, which outputs $O_{A_{i}}$ as follows:

(4) $$\begin{array}{cc} \mu_{A_{i}},\sigma_{A_{i}}= & \;e_{A}\left(I_{A_{i}};\theta_{e_{A}}\right),\\ z_{A_{i}}∼ & \;N(\mu_{A_{i}},\sigma_{A_{i}}),\\ O_{A_{i}}= & \;d_{A}\left(z_{A_{i}};\theta_{d_{A}}\right), \end{array}$$

where $e_{A}$ and $d_{A}$ represent the encoder and decoder networks of the accelerometer VAE with parameters $\theta_{e_{A}}$ and $\theta_{d_{A}}$, respectively. $z_{A_{i}}$ is then sampled from the distribution parametrised by the encoder outputs, i.e., $\mu_{A_{i}}$ and $\sigma_{A_{i}}$. The loss for the accelerometer VAE is finally defined similar to that of the silhouette module,

(5) $$L_{A}=\sum_{i}(∥O_{A_{i}}-I_{A_{i}}{∥}^{2}+KL\left(N\left(\mu_{A_{i}},\sigma_{A_{i}}\right),N\left(O,I\right)\right)).$$

**Classification Module:** The means of the latent distributions, $\mu_{S_{i}}$ and $\mu_{A_{i}}$, predicted by the two encoder models, are concatenated and passed through the classification subnetwork to output the PD vs. HC prediction as

(6) $$\begin{array}{cc} R_{i}= & concat(\mu_{S_{i}},\mu_{A_{i}}),\\ c_{i}= & f_{C}\left(R_{i};\theta_{f_{C}}\right), \end{array}$$

where $R_{i}$ is the concatenated representation, and $f_{C}$ and $\theta_{f_{C}}$ represent the classification network and its parameters, respectively. The sigmoid cross-entropy loss is used to optimise the classification objective as

(7) $$L_{C}=\sum_{i}-(y_{i}log\;p\left(c_{i}\right)+\left(1-y_{i}\right)\left(1-log\;p\left(c_{i}\right)\right)),$$

where $y_{i}$ represents the ground truth classification label.

**Missing modality:** According to Equation (6), predicting a joint representation to be passed to the classification module, requires the presence of both modalities. In the case of a missing modality during inference, due to, for example, an accelerometer not being worn for an entire day or a dropped signal on a random basis, the model would not be able to represent the features corresponding to that modality and would fail to predict the PD vs. HC label. To deal with this, we propose to estimate a representation for the missing modality using the generative capacity of our VAE models along with the representations predicted by the nonmissing modality. This will now be described in more detail.

The two VAE models in the current setting learn independent feature spaces, which are fused through the classification module. Although this fusion links the two spaces, it does not impose any constraint on the values of the learnt features. This could result in two different latent spaces and a network prone to overfitting. Furthermore, the regularisation introduced by the KL divergence loss is not imposed across modalities. Therefore, to address these limitations, we propose to add a cross-modality regularisation term to our network loss, which encourages the model to minimise the distance between the latent spaces of the two modalities. To achieve this, we minimise the cosine distance between the latent representations of the two VAE models during optimisation as

(8) $$L_{D}=\sum_{i}{\left(1-\frac{\mu_{A_{i}}\cdot \mu_{S_{i}}}{∥\mu_{A_{i}}∥\times ∥\mu_{S_{i}}∥}\right)}^{2}.$$

The final loss of the network will then be

(9) $$L=\alpha \left(L_{S}+L_{A}\right)+\beta \;L_{C}+\gamma \;L_{D},$$

where α, β, and γ represent the weights of the loss terms.

Introducing $L_{D}$ to the network loss not only has a regularisation effect on its training but also encourages the two latent spaces to be close, providing the possibility of interchanging sampled representations between the two network branches when a modality is missing. Note that, in this setting, we assume the data is fully present during training. However, we use all four losses in Equation (9) in our training, while the network is trained end-to-end. This encourages the network to keep the two latent spaces close, which prepares it for test time, where we consider a possibility for having data with missing modalities.

Thus, during inference when there is a missing modality, we estimate its representation by sampling from the latent space of the nonmissing modality, i.e.,

(10) $$\begin{array}{cc} z_{N_{i}}∼ & \;N(\mu_{N_{i}},\sigma_{N_{i}}),\\ z_{M_{i}}= & \;z_{N_{i}}, \end{array}$$

where subscripts *M* and *N* represent the missing and nonmissing modalities, respectively, such that M,N∈{A,S} and M≠N. The resulting representation is the concatenation of the missing and nonmissing representations (i.e., $z_{M_{i}}$ and $\mu_{N_{i}}$), which can then be used in our classification module to predict the PD vs. HC label. Note that, as an alternative approach, one could also consider the generative capacity of the VAE model for the missing modality itself, to generate the missing representation. We show the advantage of our cross-modality sampling approach over this, in Section 4.3.

## 4. Results

In this section, we first describe our data set in Section 4.1. We then explain the implementation details of our models in Section 4.2. We finally present our experimental results and discuss the research impact of our work in Section 4.3 and Section 4.4, respectively.

### 4.1. Data Set

The data set used in this work is based on an IoT platform [6] in a home environment, equipped with the privacy preserving RGB-D cameras, and a wearable sensor. The wearable sensor is a wrist-worn three-axis AX3 accelerometer device from Axivity [51], with a frequency of 100 Hz. The cameras are installed in a kitchen and visualise the participants from behind and from the side [49]. Each camera’s height from the floor is approximately 2 m. The distance between the camera and the participant is between 1 and 3 m. As mentioned before, due to privacy requirements, we discard the RGB and depth data after extracting the silhouette images. The accelerometer and vision sensors are synchronised using UTC timestamps. These timestamps are used to temporally align the two modalities in our preprocessing phase.

Our data set includes silhouette and accelerometer data corresponding to five heterosexual spousal pairs who are roughly age matched. Each pair consists of one person with PD and one person as the HC. From the 10 participants, 2 females and 3 males have PD, while 3 females and 2 males are the HC. The average age of the participants is 63.8 and the average time since PD diagnosis for the person with PD is 5.9 years. The duration of data recorded for PD and HC is 61.8 and 71.6 min, respectively (133.4 min in total), which provides a relatively balanced label set for our classification.

During data collection, the participants were asked to perform an unscripted cooking activity. While cooking, the participants performed a variety of actions such as walking around the kitchen, which involved their whole body movement, as well as stirring, grating, and pouring that involved more fine-grained movements of their hands. Note that while the PD-related symptoms in the latter group of activities are better captured by accelerometers, those related to the former activities are more visible in the silhouettes. For the video data, the participants were recorded separately. Thus, there is only one person at a time in the camera view. Note that the presence of multiple silhouettes in the network input is the result of averaging over multiple frames (see Figure 3).

### 4.2. Implementation Details

The silhouette module in MCPD-Net receives silhouette images of size 256×320×3. The three depth channels represent temporal averages over time intervals of 5, 150, and 250 frames. As the frequency of our silhouette extraction is, on average, 8 frames per second, these represent intervals of 0.6, 18.8, and 31.3 s, respectively. The silhouette inputs are given to the encoder of our silhouette VAE model, which contains three 3D convolutional layers with 2, 4, and 8 filters, each followed by sigmoid activations and pooling layers. Following this are two dense layers, each with 64 neurons, representing $\mu_{S_{i}}$ and $\sigma_{S_{i}}$. The sampled representation is finally given to the VAE decoder, symmetric to the encoder. Note that, due to the simplistic structure of our inputs, we did not observe any improvement in the performance of our network by increasing its depth.

The accelerometer module receives accelerometer signals corresponding to three spatial directions (*x*, *y*, *z*) for 250 time instants, which makes an input of size 250×3. The accelerometer is resampled to 10 Hz, and thus the accelerometer input represent 25-second windows of time. These are given to the accelerometer encoder, which consists of three convolutional layers with 2, 4, and 8 filters, each having three 1D convolutions for the three signals and followed by ReLU activations. The output of the last convolution is given to pooling and dense layers to predict the latent embeddings of size 64. The decoder is symmetric to the encoder. To synchronise the two modality inputs $I_{S_{i}}$ and $I_{A_{i}}$, $S_{i}$ is temporally matched with the last data point in the accelerometer sequence $I_{A_{i}}$.

Finally, the classification module consists of two dense layers of size 64 before the final binary classification layer. The hyperparameters α, β, and γ are set to 0.1, 0.1, and 1, respectively, to encode the importance of the similarity of the joint representation. The network training is performed for 5 epochs using the Adam optimiser. For all of our experiments, we perform cross-validation, where we leave one pair of subjects (one PD and one HC) out as test data, and train the network on the remaining subjects. The average number of the training and test samples across the folds is 47,079 and 11,770, which make 80% and 20% of the whole data set, respectively. We report our classification results by precision, recall, and $F_{1}$-score, all averaged across the test folds. The code was implemented in Python using Keras with the TensorFlow backend.

### 4.3. Experimental Results

We now present our results as follows. First, we discuss the quantitative results of our PD vs. HC classification. We then show the performance of MCPD-Net in dealing with missing modalities and finally present some qualitative results for the reconstruction performance of our two VAE models. In all experiments, we will be evaluating models as binary classifiers.

**PD vs. HC Classification:** We compare the performance of our proposed multimodal architecture against unimodal approaches in Table 1. We test four classification methods, namely CNN, unimodal VAE, RF, and long short-term memory (LSTM) models, on silhouette (Sil) and accelerometer (Acl) data independently.

For Sil-CNN and Acl-CNN, we use the architecture of the silhouette and accelerometer encoders in our VAE models, respectively, along with the classification module. Sil-VAE and Acl-VAE use both encoder and decoder of the silhouette and accelerometer VAEs, respectively, before the classification module. Our LSTM models have one hidden layer with 128 units. In our RF models, we perform a cross-validated parameter search for the number of trees (either 200 or 250) and the minimum number of samples in a leaf node (either 5 or 10). The Gini impurity is used to measure an optimal split. The RF and LSTM models are both trained on extracted features from the raw data. For Acl-RF and Acl-LSTM models, we extract features from the accelerometer data which are frequently used in accelerometer signal processing [52,53]. For Sil-RF and Sil-LSTM, we apply our Sil-VAE model to extract the latent features before the classification module. The results, averaged across the test folds, show that our proposed method outperforms all these unimodal approaches in all metrics. Indeed, the increase in the $F_{1}$-score, by an average of 0.25 (at least 0.18), demonstrates the ability of MCPD-Net to encode the discriminative evidence in both modalities.

We also compare the performance of our proposed architecture with four multimodal approaches in Table 2. In the first row, we present the results of CaloriNet [28] for classifying PD vs. HC. We choose this network because its architecture is based on the same two modalities as ours. Additionally, as it was designed for calorie expenditure estimation, it is relevant to our PD recognition, as PD also affects the subjects’ movement and, consequently, their energy expenditure. For fair evaluation, we replace the last regression layer of CaloriNet with our binary PD vs. HC classification layer. The results show that MCPD-Net outperforms CaloriNet. Note that the latter performs particularly poorly on the recall metric, i.e., the true positive rate or the accuracy of predicting PD in subjects with PD, which highlights the suitability of MCPD-Net for recognising PD. In the second row of Table 2, “AE without $L_{D}$” uses autoencoder (AE) models with encoder and decoder architectures similar to the ones in MCPD-Net. These, along with the classification module, are trained with only three losses, $L_{S}$ (Equation (3)), $L_{A}$ (Equation (5)), and $L_{C}$ (Equation (7)), excluding the cosine distance loss $L_{D}$ (Equation (8)). In contrast, for “AE with $L_{D}$” in the third row, the same AE models are trained using a loss that also includes $L_{D}$. Outperforming these two approaches on all metrics by our proposed method shows the benefit of the modality-specific regularisation added by the KL divergence losses in our VAE models. Finally, in the penultimate row, “VAE without $L_{D}$” shows the results of VAE and classification models with architectures similar to those of MCPD-Net, except here, $L_{D}$ is excluded from the network loss. The increase on all metrics by MCPD-Net shows the effectiveness of using our cross-modality regularisation, which helps the network generalise better to unseen data. Overall, while all methods in Table 2 outperform the previous unimodal ones in Table 1 (which again demonstrates the benefit of using multiple modalities), MCPD-Net shows an average increase in $F_{1}$-score of 0.09 over all the other multimodal approaches.

**Missing Modalities:**Table 3 and Table 4 both present our results for dealing with missing (a) silhouette and (b) accelerometer modalities. However, these tables follow two different scenarios for simulating the occurrence of a missing modality in test time. In Table 3, we randomly remove 50% of the data from the modality mentioned as missing. This is repeated 10 times and the results are reported as their average. In Table 4, we remove all of the data of the missing modality.

The last rows in each of these tables show the performance of MCPD-Net, using Equation (10) for estimating the missing representations. We compare this against the results of our best performing unimodal models, which correspond to the available modality, i.e., ‘Acl VAE’ and ‘Sil VAE’, in the first rows of Table 3 and Table 4. Note that in Table 3, 50% of the data are presented to the network with a missing modality, and 50% of the data are presented without any missing modality. In this case, when a modality is missing, unimodal models “Acl VAE” and “Sil VAE” are used to predict the classification labels. For the other half of the data, in which both modalities are present, MCPD-Net is used to predict the classification labels. Outperforming both these unimodal models shows that, even if a modality is missing during test time, whether all of the data are missing or 50% of the data are missing, MCPD-Net still benefits from what is learned from both modalities during training. In the second and third rows of Table 3 as well as Table 4, we also compare MCPD-Net against “AE with $L_{D}$” and “VAE without $L_{D}$”. These are multimodal models capable of dealing with missing modalities in our PD classification context. We do not consider other models such as CaloriNet, as their architectures are not designed to deal with a missing modality, i.e., their classification requires the presence of both modalities. For “AE with $L_{D}$”, the nonmissing module is first used to predict its own latent representation during inference. This same prediction is then used for representing the missing modality. For “VAE without $L_{D}$”, in contrast, the generative capacity of the VAE model corresponding to the missing modality is tested in generating the representation required for classification. This is done by first sampling a latent vector from a standard normal distribution and then feeding it through the decoder and encoder networks of the missing modality, respectively. Note that, “VAE without $L_{D}$” has not been trained with $L_{D}$; thus, its only regularisation is due to minimising the KL divergence between the encoder output and standard normal distribution. The results show that, in both missing data scenarios, our proposed method outperforms both these multimodal approaches on the $F_{1}$-score, demonstrating the advantage of sampling and exchanging representations across modalities, compared to using the same nonmissing predictions or only using the missing modality to estimate the missing feature. We also find that, especially in the whole modality missing scenario, our network achieves a better performance when silhouette is missing, compared to missing accelerometer, with $F_{1}$-score of 0.62 vs. 0.51. This shows that, via the joint learning, the accelerometer module has been able to encode more discriminative PD symptoms, while also capturing a good estimation of the silhouette representations. Note that the relatively low recall for all approaches (including MCPD-Net) in Table 4b shows the disadvantage of missing accelerometer for PD recognition in PD subjects. However, overall, MCPD-Net outperforms all the other approaches by an average increase in $F_{1}$-score of 0.17 (0.22 and 0.12 for missing silhouette and accelerometer, respectively).

**Figure 4:**
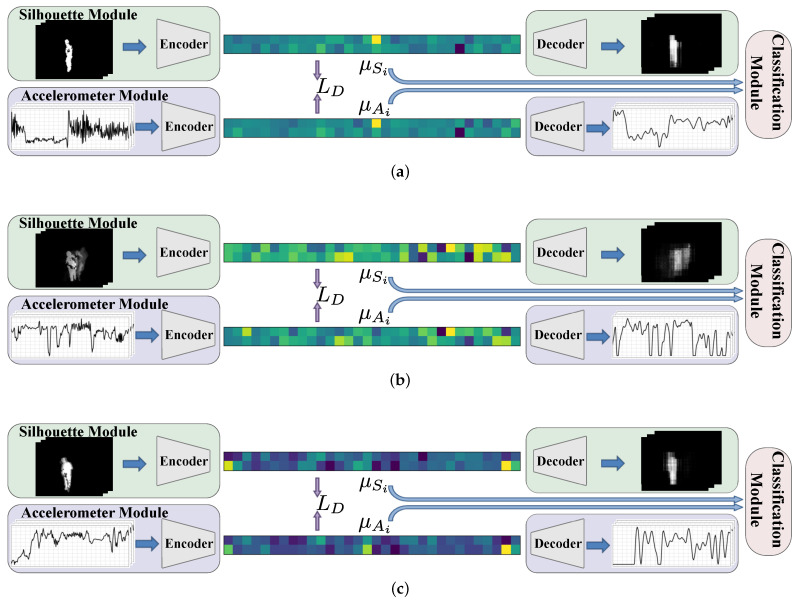
Qualitative results for the similarity between the learnt representations in the two latent spaces, for three examples in (**a**–**c**), respectively. The first and second rows in each example show the silhouette and accelerometer modules, and the similarity between their extracted features demonstrates the effectiveness of the $L_{D}$ loss.

To further analyse the performance of our proposed method for the similarity that is learned between the latent spaces of the two VAE models, we illustrate our network for three examples in Figure 4a–c. Each of these figures is a simplified version of Figure 2, presenting the two branches of our proposed architecture for silhouette and accelerometer modalities as well as their connection through the classification module. However, our focus here is on the two colour-coded vectors in between the encoder and decoder of the silhouette and accelerometer modules. These are the mean of the latent distribution in the silhouette module, i.e., $\mu_{S_{i}}$ (in Equation (2)), and the corresponding values in the accelerometer module, i.e., $\mu_{A_{i}}$ (in Equation (4)). Note that $\mu_{S_{i}}$ and $\mu_{A_{i}}$ are representations corresponding to a pair of data points in the silhouette and accelerometer input domains, respectively, which are jointly fed through the two VAE encoder models. These inputs are shown on the left to the encoders. The reconstructed outputs are also shown on the right to the decoders.

These results visualise the similarity between $\mu_{S_{i}}$ and $\mu_{A_{i}}$ feature vectors extracted from the two branches of the network. This is due to the use of $L_{D}$ in the network loss during training. In other words, this similarity is learned during training. However, during test time, it provides a possibility for our model to use the latent space of the present modality to generate a representation for the missing modality.

**Figure 5:**
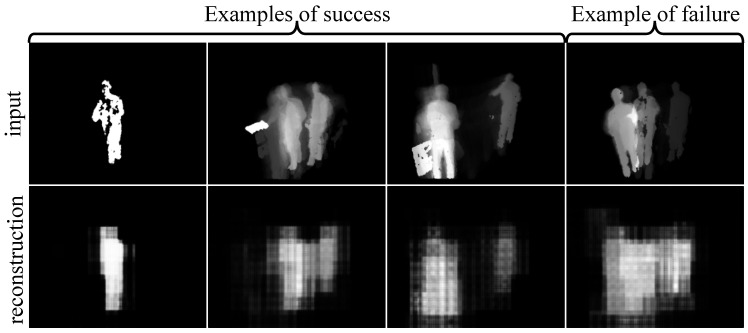
Successful (**first three columns**) and failure (**last column**) examples for the silhouette VAE to reconstruct both spatial and temporal information in the input. The first and second rows show the silhouette inputs and their corresponding reconstructions, respectively.

**Silhouette and Accelerometer Modules:** We finally present some qualitative results to show the performance of our network in reconstructing its inputs. Figure 5 presents three examples of success from the silhouette module in the first three columns, and an example of failure in the last column. The first and second rows show the silhouette inputs and their corresponding reconstructions, respectively. As seen in the success cases, both spatial and temporal information in the input have been successfully reconstructed. More specifically, the model has been able to reconstruct the silhouette in the correct spatial location and capture the silhouette displacement during time. It has also removed the noise in the input. In the failure case, however, the reconstructed output by the silhouette module incorrectly shows the subject moving around. This could be due to overfitting on such examples during training.

**Figure 6:**
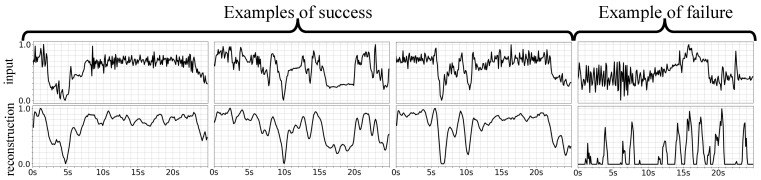
Successful (**first three columns**) and failure (**last column**) examples for the accelerometer VAE to capture the input signal pattern and remove its noise. The *x* axis shows time in seconds, while the *y* axis represents the acceleration signal on one of the spatial directions. The first and second rows show the accelerometer inputs, and their corresponding reconstructions, respectively.

Similarly, Figure 6 presents three examples of success by the accelerometer VAE model in the first three columns, and an example of failure in the last column. The first and second rows show the accelerometer input and their corresponding reconstructed output signals, respectively, both normalised between 0 and 1. Note that the input accelerometer is the raw signal, while its reconstruction is the output of the network activation. These two signals are normalised for the purpose of visualisation using $s^{norm}=\frac{s-min(s)}{max(s)-min(s)}$, where *s* and $s^{norm}$ represent the original and normalised signals, respectively. The *x* axis shows time in seconds, while the *y* axis represents the acceleration signal. The examples of success demonstrate good reconstruction performance by the accelerometer module, as the model has not only captured the pattern of the input signal but also smoothed its noise. In the failure example, though, the model shows a poor reconstruction performance, potentially due to the high level of noise in the input.

### 4.4. Discussion

Evaluating PD in free living conditions has several advantages, such as reducing the Hawthorne effect [54] of observation by a clinician on behaviour/symptoms and improving the ecological validity of outcome measures used in clinical trials and practice to measure symptom progression in PD. It also provides the possibility for a continuous monitoring of the person with PD, while they are in their own home, recording rare events such as falls, activities which occur more naturally away from the clinic environment (such as hobbies) and capturing the hour-by-hour symptom fluctuations of this condition. The research community has consequently shown increasing interest in PD evaluation via automatic approaches in home settings. Many models have been trained on sensor data obtained from PD subjects in such settings to classify or measure the severity of PD symptoms with promising results. However, in these automatic approaches, some aspects of the assessment are neglected. As an example, while the specialists in clinical settings would consider the whole body movements to get an impression of how severe the symptoms are, the existing automatic machine-learning-based approaches frequently produce their outcome measures based on data collected from a single sensor. As a result, the symptoms captured are limited to specific body parts depending on what and where the sensor is applied. For example, if a wrist-worn sensor is used, it can only capture those symptoms that affect the wrist movements. Similarly, a vision sensor would only capture the appearance of the subject from a single viewpoint, which might cause missing important body parts such as hands.

Thus, an automatic approach would benefit from expanding its input domain to capture a more general overview of the symptoms. We propose that such expansion of the input in a multimodal approach would increase the sensitivity of symptom evaluation and, therefore, would be especially effective for evaluating PD in naturalistic setting, with subjects who are well medicated and present mild symptoms, or similarly, for an early diagnosis of PD, where the symptoms are more challenging to detect, even by neurology specialist clinicians.

In this work, we made progress toward such an evaluation approach by combining two input modalities. We use wrist-worn accelerometer and vision sensors to capture these two modalities. Our machine learning method leverages the potential of the VAE models in encoding the dynamics of the performed activities in both spatial and temporal domains and generating robust features per data modality. The correlation between the input modalities is captured by fusing them through the classification module. In our results, discussed in Section 4.3, we objectively demonstrate that our method outperforms several unimodal approaches, which confirms the advantage of a multimodal approach for evaluating PD. To present further evidence for the superiority of our proposed method, we also show that it outperforms other multimodal approaches.

Another aspect of our work that distinguishes it from other related works is its resiliency to missing modalities. An IoT platform with multiple sensors used for continuous data collection is prone to technical faults that may result in missing data. Privacy or cost factors may also prevent recording of certain data types in some areas of a home such as bedrooms or bathrooms. Considering these possibilities, we design our method to be able to deal with such missing modalities during inference. We specifically use the similarity between the latent spaces of the two modalities to generate a feature for the missing modality. In Section 4.3, we discussed the high classification results of our approach, compared to other multi- and unimodal methods, in more detail. To the best of our knowledge, we are the first work using multiple modalities to recognise PD vs. HC in home environments, while resilient to a missing modality.

## 5. Conclusions

In this work, we proposed MCPD-Net, a multimodal deep learning model that learns joint representations of different modalities for a classification task. We evaluated our proposed model on data collected of people with and without Parkinson’s disease that were performing cooking activities in a home environment. During the data collection, subjects were wearing a wrist-worn wearable accelerometer, while the room contained a privacy-preserving camera that extracted image silhouettes.

The novelty of our method, in the context of PD assessment, is based on using an IoT platform to collect data from multiple sensors. The use of the two data modalities in our approach results in capturing a wide range of PD symptoms from different body parts. Moreover, our analysis approach is based on the data from subjects who are performing cooking activities, while the PD subjects are well medicated. This shows the value of our work to be used in naturalistic settings to capture activities of daily living that occur away from a laboratory environment. Another novelty is the ability of our method in dealing with missing modalities, which is a common issue with “in the wild” deployments of smart home systems. In terms of the machine learning approach, we proposed the use of VAE models to learn robust features per modality for an effective PD classification. We also introduced a loss function to our network architecture, which enables our method to learn a similarity between the latent spaces across modalities. Using this learnt similarity, we propose to use the generative capacity of the VAE model of the available modality during test time to generate features for the missing modality.

Using both the accelerometer and silhouette data, we demonstrated that our proposed model is able to outperform existing methods at the task of predicting whether or not the subject has Parkinson’s disease, with an average increase in $F_{1}$ score of 0.25 and 0.09, compared to unimodel and other multimodal approaches, respectively. Furthermore, we quantitatively and qualitatively demonstrated our model’s ability to perform with missing modalities during the inference stage, achieving an average increase in $F_{1}$ score of 0.17 over unimodal approaches when a modality is missing.

For future work, we aim to extend this work by collecting a larger data set in which the participants stay in a house equipped with multiple sensors for a longer duration. We aim to record the participants while performing clinical tests and scripted activities, and more importantly, over long periods of free living. We will use this data to build novel models for monitoring the progression of the disease and measuring different PD symptoms [55].

## Figures and Tables

**Table 1:** MCPD-Net vs. unimodal architectures. Our proposed multimodal method outperforms all of the other approaches.

		Precision	Recall	$F_{1}$-Score
**Silhouette (Sil)**	**CNN**	0.17	0.40	0.24
	**VAE**	0.49	0.49	0.47
	**RF**	0.46	0.39	0.41
	**LSTM**	0.45	0.40	0.41
**Accelerometer (Acl)**	**CNN**	0.53	0.45	0.44
	**VAE**	0.63	0.55	0.44
	**RF**	0.59	0.45	0.43
	**LSTM**	0.58	0.47	0.42
**MCPD-Net**		**0.71**	**0.77**	**0.66**

**Table 2:** MCPD-Net vs. other multimodal architectures. This demonstrates the superiority of MCPD-Net, due to its VAE models and cross-modality regularisation $L_{D}$.

	Precision	Recall	$F_{1}$-Score
**CaloriNet [28]**	0.65	0.48	0.50
**AE without $L_{D}$**	0.69	0.56	0.58
**AE with $L_{D}$**	0.69	0.58	0.61
**VAE without $L_{D}$**	0.61	0.67	0.58
**MCPD-Net** **_(VAE with *L_D_*)_**	**0.71**	**0.77**	**0.66**

**Table 3:** Performance of MCPD-Net when 50% of the silhouette and accelerometer data are missing. This demonstrates an overall improvement by our proposed method.

	Precision	Recall	*F*_1_-Score
**(a) Missing Sil (Only Using Acl)**			
**Acl VAE** **_(unimodal)_**	0.69	0.66	0.58
**AE with $L_{D}$** **_(multimodal)_**	0.61	0.40	0.46
**VAE without $L_{D}$** **_(multimodal)_**	0.63	0.62	0.57
**MCPD-Net**	**0.70**	**0.77**	**0.64**
**(b) Missing Acl (only Using Sil)**			
**Sil VAE** **_(unimodal)_**	0.57	**0.63**	0.59
**AE with $L_{D}$** **_(multimodal)_**	**0.67**	0.42	0.48
**VAE without $L_{D}$** **_(multimodal)_**	0.58	0.61	0.55
**MCPD-Net**	0.63	**0.63**	**0.63**

**Table 4:** Performance of MCPD-Net when silhouette and accelerometer data are completely missing. This demonstrates an overall improvement by our proposed method.

	Precision	Recall	*F*_1_-Score
**(a) Missing Sil (Only Using Acl)**			
**Acl VAE** **_(unimodal)_**	0.63	0.55	0.44
**AE with $L_{D}$** **_(multimodal)_**	0.20	0.22	0.20
**VAE without $L_{D}$** **_(multimodal)_**	0.63	0.56	0.55
**MCPD-Net**	**0.70**	**0.77**	**0.62**
**(b) Missing Acl (only Using Sil)**			
**Sil VAE** **_(unimodal)_**	0.49	0.49	0.47
**AE with $L_{D}$** **_(multimodal)_**	0.30	0.25	0.23
**VAE without $L_{D}$** **_(multimodal)_**	0.56	**0.54**	0.46
**MCPD-Net**	**0.60**	0.49	**0.51**

## Data Availability

The data used in this study is not publicly available, as it contains restricted sensitive personal data.
